# The Inhibitory Role of B7-H4 in Antitumor Immunity: Association with Cancer Progression and Survival

**DOI:** 10.1155/2011/695834

**Published:** 2011-10-13

**Authors:** Changjun He, Haiquan Qiao, Hongchi Jiang, Xueying Sun

**Affiliations:** ^1^The Hepatosplenic Surgery Center, Department of General Surgery, The First Affiliated Hospital of Harbin Medical University, Harbin 150001, China; ^2^Department of Molecular Medicine and Pathology, Faculty of Medical and Health Sciences, The University of Auckland, Auckland 1005, New Zealand

## Abstract

B7-H4 is one of the most recently identified members of B7 superfamily of costimulatory molecules serving as an inhibitory modulator of T-cell response. B7-H4 is broadly expressed in human peripheral tissues and inducibly expressed in immune cells. The expression of B7-H4 has been observed in various types of human cancer tissues, and its soluble form has been detected in blood samples from cancer patients. However, its precise physiological role is still elusive, as its receptor has not been identified and the expression levels are not consistent. This paper summarizes the pertinent data on the inhibitory role of B7-H4 in antitumor immunity and its association with cancer progression and survival in human patients. The paper also discusses the clinical significance of investigating B7-H4 as potential markers for cancer diagnosis and prognosis, and as therapeutic targets.

## 1. Introduction

Activation of T lymphocytes requires two independent but mandatory signals. The first signal requires recognition of the major histocompatibility complex (MHC)/antigen on antigen presenting cells (APCs) and corresponding antigen-specific T-cell receptor (TCR) on T cells. On the other hand, the second signal is delivered by the binding of costimulatory molecules and their receptors/ligands. In the absence of the costimulatory signal, the ligation of TCR with the MHC/antigen complex results in disfunction or anergy of T cells. The typical costimulatory signals are rendered by the molecules of the “classic” B7 family including CD80, CD86, and their receptor CD28 and cytotoxic T lymphocyte antigen (CTL-4), which could provide positive and/or negative costimulatory signals in initiating T-cell response. Recently, several B7 homologues have been identified, including B7-H1, B7DC, B7-H2, B7-H3, B7-H4, and B7-H6. B7-H4 (also known as B7x or B7S1) is among the most recently identified members of the B7 superfamily. It is broadly expressed in many human tissues and cells, and is shown to regulate adaptive immune response by inhibiting the proliferation, activation, and cytokine production of T cells, and host innate immune response by suppressing growth of neutrophil progenitors. It is also expressed in many types of human cancers, and has been used as a negative prognostic indicator for many human tumors. Therefore, B7-H4 represents a novel frontier of investigation for understanding the molecular regulation of the immune system and targeting B7-H4 may help to overcome the inhibitory immune network in tumor environments. This paper discusses the inhibitory role of B7-H4 in antitumor immunity, and its association with cancer progression and survival in human patients. It also discusses the clinical significance of investigating B7-H4 as potential markers for cancer diagnosis, and prognosis, and as therapeutic targets.

## 2. Structure and Expression Pattern of B7-H4

B7-H4 was identified by DNA sequence homology with other molecules of the B7 family in 2003 by three laboratories, which designated three different names to the same molecule, that is, B7S1, B7-H4, and B7x, respectively [1–3], but now B7-H4 has been most widely used. B7-H4 is a type I transmembrane protein and has 20–30% amino acid homology in the extracellular portion with other B7 family members. The mouse and human amino acid sequences of B7-H4 share approximately 87% amino acid identity [2]. B7-H4 mRNA is widely expressed in human peripheral tissues, including lung, testis, pancreas, prostate, placenta, uterus, skin, muscle, intestine, stomach, kidney, liver, heart, brain, and ovary [1–3]. However, its protein expression on tissues seems to be limited [2, 4]. Initially, B7-H4 expression was observed in cancer cells of colon, prostate, lung, and fibrosarcoma [3, 5], and human ovarian and lung cancer tissues [4]. Subsequent studies from different laboratories have demonstrated that the expression of B7-H4 mRNA and protein was detected in all of the 23 melanoma cell lines [6], 5 gastric cancer cell lines [7], and 6 non-small-cell lung cancer cell lines [8]. To date, B7-H4 expression has been found in many different types of human cancer tissues, and soluble B7-H4 has also been detected in blood samples from cancer patients. The expression pattern of B7-H4 in human cancer tissues and its clinical significances will be discussed in Section 4. 

B7-H4 is not expressed in naïve T and B cells, but after stimulation by interleukin-6 (IL-6) and IL-10, B7-H4 is inducibly expressed in APCs, including dendritic cells (DCs), monocytes and macrophages [1–3]. On the other hand, the DC-differentiation cytokines, granulocyte/macrophage colony-stimulating factor (GM-CSF) and IL-4, decrease the expression of B7-H4 in these cells [9–11]. However, interferons (INFs) appear to have minimal effects on the induction of B7-H4 expression [9–11].

In human ovarian cancer, tumor-associated regulatory T (Treg) cells trigger macrophages to produce IL-6 and IL-10, and these cytokines in turn stimulate APCs to express B7-H4 in an autocrine and/or paracrine manner [9]. High levels of IL-6 and IL-10, but not GM-CSF and IL-4, are detected in the ovarian tumor microenvironment. Therefore, this dysfunctional cytokine network in the tumor microenvironment may enable APCs to express B7-H4. Interestingly, IL-4, IL-6, IL-10 and GM-CSF have no regulatory effects on the expression of B7-H4 on tumor cells, indicating that the expression of B7-H4 in tumor cells may be functionally distinct and differently regulated compared with APCs [9, 12].

To date, the receptor of B7-H4 has not yet been identified. B and T lymphocyte attenuator (BTLA) was initially proposed to be the receptor for B7-H4 [5], but further studies have not supported this proposal, as BTLA has not shown to directly bind to B7-H4 but may influence the appearance of an unknown receptor for B7-H4 on the Th1 cell surface [13–15]. 

## 3. Negative Effects of B7-H4 on Antitumor Immunity

### 3.1. Adaptive Immunity

B7-H4 inhibits the activation, proliferation, clonal expansion of CD4^+^ and CD8^+^ T-cells, thus suppressing the production of cytokines (IL-2, IFN-*γ*), and generation of alloreactive cytotoxic T lymphocytes (CTLs) by arresting the cell cycle in an *in vitro* T-cell activation assay [1, 2, 5]. B7-H4 expressed on the surface of surrogate APCs also inhibits the proliferation of T cells [2, 5]. *In vivo* blockade of endogenous B7-H4 by a specific mAb promoted T-cell response, indicating that B7-H4 plays an inhibitory role in T-cell activation [2]. The inhibitory effects of B7-H4 on T-cell activation and proliferation are also supported by the finding that acute lymphopenia-induced homeostatic proliferation of T cells promotes antitumor immunity. However, these cells display a severe deficit in the expression of B7-H4 as they show lower suppression by a specific Ab against B7-H4 and fail to produce IL-10 [16]. B7-H4-deficient Balb/c mice mounted mildly augmented Th1 responses and displayed slightly lower parasite burdens upon Leishmania major infection compared to the wild-type mice, indicating that B7-H4 could inhibit Th1 response against infection [17]. However, the lack of B7-H4 did not affect hypersensitive inflammatory responses in the airway or skin that are induced by either Th1 or Th2 cells. Likewise, B7-H4-deficient mice developed normal CTL reaction against viral infection [17]. These results suggest that B7-H4 may be one of multiple negative cosignaling molecules that collectively provide a fine-tuning mechanism for T-cell-mediated immune responses [18]. 

 There is no direct evidence of a barrier function for B7-H4, although it is variably glycosylated in tumor-specific patterns, suggesting that glycosylation may be a potential mechanism for modulating interaction of CTLs with tumor cells [19]. A physical blockade would complement the ability of B7-H4, when ligated to its unknown receptor on T cells, to inhibit cytokine secretion, and proliferation of T cells predominantly through cell cycle arrest [2].

The effects of B7-H4 on B cells have not been investigated. However, enhanced B7-H4 expression on B cells infected with Epstein-Barr virus (EBV) increased the levels of intracellular reactive oxygen species (ROS), induced the expression of Fas ligand, and subsequently led to Fas-mediated and caspase-dependent apoptosis in association with increased release of cytochrome c, apoptosis-inducing factor (AIF), and EndoG from the mitochondria [20]. In a subsequent study by the same group, engagement of B7-H4 significantly reduced cell growth of EBV-positive lymphoma cells, resulting in cell cycle arrest at G0-G1 phase via downregulation of CDK4/6, CDK2, cyclin E/D expression, phosphor-AKT, and phosphor-cyclin E and upregulation of p21 expression [21]. These results suggest that B7-H4 may be a potential target for EBV-positive lymphoma therapy. Although not investigated, these studies may also imply that B7-H4 could inhibit proliferation and activation, and induce apoptosis of B cells, thus impairing the production of immunoglobulins and contributing to the suppression of adaptive immunity.

In addition, it has been demonstrated that tumoral B7-H4^+^ macrophages and CD4^+^CD25^+^FoxP3^+^ Treg cells suppressed tumor-associated antigen-specific T-cell immunity [10]. The tumor-associated macrophages spontaneously produce chemokine CCL22 to mediate Treg cell trafficking into tumors, and Treg cells induce the expression of B7-H4 on APCs and macrophages [10]. It has been shown that Treg cells induced macrophages to spontaneously produce IL-10 and IL-6, which in turn stimulated B7-H4 expression on macrophages in an autocrine manner through IL-10 and IL-6 [9]. The two studies suggest that Treg cells may convey their suppressive activity to APCs through B7-H4 induction [9]. 

### 3.2. Innate Immunity

To date, there has been only one published study [22], which has investigated the role of B7-H4 in innate immunity. It has been shown that the inhibitory effect of B7-H4 on innate immunity was mediated through controlling the growth of neutrophils [22]. B7-H4 knockout mice were more resistant to infection by Listeria monocytogenes than their littermates, suggesting that B7-H4 plays an inhibitory role on innate immunity. Further studies have shown that more neutrophils were observed in peripheral organs of B7-H4 knockout mice than their littermates but their bactericidal functions remained unchanged. *In vitro*, B7-H4 inhibited the growth of bone marrow-derived neutrophil progenitors, suggesting an inhibitory function of B7-H4 in neutrophil expansion. As augmented innate resistance is completely dependent on neutrophils, even in the absence of adaptive immunity, the results indicate that B7-H4 serves as a negative regulator of the neutrophil response to infection, and provides a new target for manipulation of innate immunity.

### 3.3. Cancer Immunity

B7-H4 has been found to be expressed at the mRNA and protein levels in many types of human cancers and negatively correlate with poor prognosis (Refer to Section 4). Expression of B7-H4 in human tumors is most likely due to aberrant regulation of posttranscription in tumors, since its cell surface protein expression is rare in normal human tissues, though abundant B7-H4 mRNA is detected [18]. B7-H4 was preferentially expressed in nondividing tumor cells from human gliomas and medulloblastomas, and in a subset of brain tumor stem-like CD133^+^ cells [23]. The CD133^+^ cell-initiated glioblastomas showed a higher proliferation index than CD133^−^ cell-induced glioblastomas in immune-deficient mice [23]. 

Increased B7-H4 expression in tumor cells correlated with decreased cell apoptosis and enhanced outgrowth of tumors in several models, including the severe combined immunodeficiency (SCID)/Beige xenograft outgrowth model [22]. B7-H4 has also been shown to be extensively and variably N-glycosylated, which may serve as a “barrier” mechanism to evade immunosurveillance [22]. As suggested by Yi and Chen, the role of B7-H4 in tumor progression may be to transform precancerous cells and then protect them from immunosurveillance [18]. In addition, one study has shown that overexpression of B7-H4 promoted tumorigenesis of ovarian cancer in immunodeficient mice by increased proliferation rate, cell adhesion, migration, and invasion [24], implying that B7-H4 might have a direct effect on tumorigenesis independent of immunity. In another study, overexpression of B7-H4 on normal cells resulted in malignant cellular transformation of epithelial cells, perhaps by protecting the pretransformed cells from apoptosis, as siRNA knockdown of B7-H4 on tumor cell lines *in vitro* led to increased apoptosis [19]. However, the direct effect of B7-H4 on tumorigenesis has been only demonstrated in the above two studies, thus the exact mechanisms need further investigation. 

In the tumor microenvironment, in addition to tumor cells, tumor-infiltrating macrophages [9, 10] and endothelial cells of small blood vessels [12] have also been found to constitutively express B7-H4. B7-H4 was highly expressed in tumor-associated macrophages in the ascites of ovarian cancer patients and contributed to tumor progression [10]. B7-H4 blockade by antisense oligonucleotides restored the function of macrophages to stimulate T cells and led to tumor regression *in vivo* [10, 11]. 

## 4. B7-H4 Expression in Human Cancers and Its Significance

In the present paper, the clinical data in support of the possible function of B7-H4 in antitumor immunity come from 26 retrospective analyses on 13 types of human cancers including the most common ones, that is, cancers of ovary, esophagus, kidney, stomach, liver, lung, colon, pancreas, breast and prostate, and melanoma. All relevant studies on the expression of B7-H4 on human cancer tissues or levels of soluble B7-H4 in human blood samples and the clinical significance are summarized in Table 1 [4–9, 12, 19, 23, 25–41]. A negative correlation between B7-H4 expression and T-cell infiltration has been reported [25, 32, 34]. However, such correlation was not observed in a study on melanoma [6]. 

 The expression of B7-H4 has been most widely studied in ovarian cancer. To date, ten studies have investigated the expression of B7-H4 in ovarian cancer tissues and/or the level of soluble B7-H4 in blood samples from the ovarian cancer patients [4, 9, 19, 23, 28–30, 35–38]. The positive B7-H4 expression rates in ovarian cancer tissues range from 9 to 100% as shown by immunohistochemistry. Most of the studies have revealed the correlation between expression levels of B7-H4 and survival, pathological types, or tumor TNM staging. The levels of soluble B7-H4 in blood correlate with tumor stage, poor prognosis, and pathological types, indicating that B7-H4 may be a potential diagnostic marker and a prognostic predictor for ovarian cancer. However, one study did not show the similar correlation between soluble B7-H4 levels in blood and other diagnostic markers for ovarian cancer patients [27]. 

Breast cancer is the second most studied cancer for B7-H4 expression. To date, six studies have investigated the expression of B7-H4 in human breast cancer tissues [19, 30, 32, 33, 37], but two of which lack detailed data. In one study, 193 primary breast tumors and 246 metastatic breast tumors were examined by immunohistochemistry and the B7-H4 positive expression rates were as high as 95.4% in primary tumors and 97.6% in metastatic tumors, and the increased expression of B7-H4 correlated with negative progesterone receptor and HER-2/neu status [33]. Similarly, the other two studies have demonstrated a positive expression rate of B7-H4 mRNA and protein at 100%, determined by reverse transcription polymerase chain reaction (RT-PCR) [41] and immunohistochemistry [19], respectively. 

In two studies on lung cancer, 31% [4] and 43% [8] of lung cancer tissues were found to express B7-H4 detected by immunohistochemistry, respectively. In a study with 259 cases of renal cell carcinoma (RCC), 59.1% of the cancer tissues had B7-H4 protein expression [12]. However, the B7-H4 positive expression rate was found to be only 17.6% in 102 cases of early-stage RCC (T1), and B7-H4 expression did not correlate with age, gender, TNM stage, lymphovascular invasion, or nuclear grade, but correlated with cancer recurrence and negatively correlated with survival [26]. 75.5% (71/94) of gastric cancer tissues were found to express B7-H4 mRNA [7], but the B7-H4 protein positive expression rate detected by immunohistochemistry dropped to 44.9% in another study with 156 cases of gastric cancer [27]. In one study with 24 cases of gastric cancer, the positive rate was as low as only 12.5%. Although not widely investigated, over 90% of the tissues from melanoma [6], pancreatic ductal adenocarcinoma [31], uterine cancer [30, 34], esophageal squamous cell carcinoma [25], and prostate cancer [5] expressed B7-H4, shown by immunohistochemistry. Although one study has reported that 63.6% (14/22) of colon cancer tissues expressed B7-H4 [30], the expression of B7-H4 was found to be less consistent [37, 41]. B7-H4 was found to be expressed in 100% (34/34) of Brenner tumors [40]. Although Brenner tumors are of benign feature, this report is exceptionally included in Table 1.

Soluble B7-H4 was detected in blood samples from patients of ovarian cancer, RCC, colon cancer, breast cancer, lung cancer, and prostate cancer [8, 28, 29, 35–37, 39]. These studies indicate that serum B7-H4 may be a useful marker for diagnosis and prognosis, but the mechanism of production and the function of soluble B7-H4 remains unknown.

## 5. Potential of B7-H4 in Clinical Application

Because of the higher expression of B7-H4 in cancer tissues compared with corresponding normal tissues and its close correlation with stage, pathological types and biological behavior of tumors, and survival of cancer patients, we should pay attention to the potential diagnostic and prognostic capacities of B7-H4 for identifying cancer, determining pathologic variables, and predicting response to treatment and survival. We believe that B7-H4 could become potent tools to add to the oncologist's toolbox for early diagnosing cancer, monitoring the efficacy of treatments and predicting the prognosis. This may be possible when the expression patterns of B7-H4 have been investigated on a larger number of samples from different types of cancers and from multiple centers.

Given that B7-H4 is highly expressed in almost all the examined cell lines from cancers of colon, prostate, lung, and stomach, and fibrosarcoma and melanomas [3–8] and in various human cancer tissues (Table 1), it could be hypothesized that the expression of B7-H4 represents a mechanism of downregulating antitumor immunity, particularly T-cell response, at the level of the effector cells [5]. This paradigm calls for the development of new strategies for tumor immunotherapy by targeting B7-H4 [42]. B7-H4 inhibits T-cell function [1–3, 5], indicating that B7-H4-positive tumor cells have an advantage over the B7-H4-negative tumor cells by downregulating T-cell-mediated antitumor immunity. Consequently, the blockade of tumor-associated B7-H4 could offer a new therapeutic opportunity for enhancing antitumor immunity. Efficient neutralizing antibodies specific for human B7*‑*H4 are not yet available. Small interfering RNA (siRNA) [19] and antisense oligonucleotides specific for B7-H4 [10, 11] have been used to block B7-H4 expression. Blocking the expression of B7-H4 in tumor-associated macrophages disabled their suppressive capacity, enabled tumor-associated antigen- (TAA-) specific effector T cells function, and suppressed tumor growth in human ovarian cancer xenografts [10, 11]. In addition, the expression of B7-H4 on endothelial cells of tumor vasculature has also been observed in RCC tissues [12]. Although the mechanism accounting for what signals trigger B7-H4 expression in tumor vessels remains unknown, one most likely source could be the tumor microenvironments. Tumor blood vessels are distinct from normal resting blood vessels, and can be selectively destroyed without significantly affecting normal vessels. Therefore, blockade and/or destruction of tumor vasculature-associated B7-H4 might provide a dual beneficial therapy, that is, enhancement of T cell-mediated antitumor immunity and destruction of tumor vessels. 

## 6. Conclusions and Future Prospects

Recent data indicate that B7-H4 functions in peripheral tissues to negatively regulate immune responses in target organs. While its broad distribution is observed at mRNA level, limited expression at the protein level suggests that tight control of B7-H4 is imposed at posttranscriptional level. Receptor identification remains the manifest topic and is critical for understanding the role of B7-H4, as it is certainly essential to understand the complex role, but continues to be difficult due primarily to low receptor/ligand affinities. Therefore, more studies are required to seek and identify the receptor for B7-H4. Increased B7-H4 expression in tumor tissues and high levels in blood samples of cancer patients represent a realistic opportunity to design novel immunotherapeutic approaches by regulating the immune response through manipulating the expression of B7-H4 and/or its receptor. B7-H4 can also serve as a useful biomarker for cancer diagnosis and prognosis prediction, when its expression patterns have been further investigated. 

## Figures and Tables

**Table 1:** Relevant clinical studies investigating B7-H4 in samples from human cancer patients.

Author [ref.]	Journal	Year	Type of cancer	No. of samples	Methods	Positive rate (%)	Significances
Chen et al. [25]	Cancer Immunol Immunother	2011	Esophageal squamous cell carcinoma	112	IHC	95.5%	Correlation with gender, distant metastasis, TNM stage; reverse correlation with densities of CD3^+^ and CD8^+^ T cells, and survival

Jung et al. [26]	Korean J Urol	2011	Renal cell carcinoma	102	IHC	17.6% (early-stage T1)	No correlation with age, gender, TNM stage, lymphovascular invasion or nuclear grade; correlation with recurrence; reverse correlation with survival

Quandt et al. [6]	Clin Cancer Res	2011	Melanoma	29	IHC	96.6% (primary), 89.7% (metastatic)	Reverse correlation with survival; no correlation with CD8^+^ T-cell infiltration

Arigami et al. [7]	J Surg Oncol	2010	Gastric cancer	94	RT-PCR	75.5%	Reverse correlation with survival

Jiang et al. [27]	Cancer Immunol Immunother	2010	Gastric cancer	156	IHC	44.9%	Reverse correlation with survival

Anderson et al. [28]	J Natl Cancer Inst	2010	Ovarian cancer	34	ELISA	—	No correlation with diagnosis markers

Qian et al. [30]	Clin Exp Med	2010	11 types of cancer*	289	IHC	Overall 52.9% (details refer to the article)	Correlation with stages

Yee et al. [40]	Histopathology	2010	Brenner tumor	34	IHC	100%	Higher proportion of expression than CA-125 and CEA

Oikonomopoulou et al. [29]	Br J Cancer	2008	Ovarian cancer	98	ELISA	—	Useful in predicting short-term (1-year) survival, time to progression after chemotherapy

Awadallah et al. [31]	Pancreas	2008	Pancreatic ductal adenocarcinoma	36	IHC	91.7%	More powerful than p53; potential diagnostic use

Thompson RH et al. [39]	Cancer Res	2008	Renal cell carcinoma	101	ELISA	52.5%	Correlation with stage; a potential serum marker for diagnosis and prognosis

Mugler et al. [32]	Appl Immunohistochem Mol Morphol	2007	Breast cancer	—	IHC	—	Correlation with invasive ductal carcinoma and reduced T-lymphocytes infiltration

Kryczek et al. [9]	Cancer Res	2007	Ovarian carcinoma	103	IHC	—	Correlation with Treg cell numbers

Miyatake et al. [34]	Gynecol Oncol	2007	Uterine endometrioid adenocarcinoma	90	IHC, WB	100%	Correlation with high risk of uterine endometrioid adenocarcinoma; reverse correlation with T-cell infiltration

Simon et al. [35]	Gynecol Oncol	2007	Ovarian cancer	251	ELISA	48% (Stage I) 55% (Stage II) 67% (Stage III)	Correlation with poor prognosis

Simon et al. [36]	Gynecol Oncol	2007	Ovarian cancer	68	ELISA	100%	A promising marker for early detection of ovarian cancer

Zang X et al. [5]	Proc Natl Acad SCI USA	2007	Prostate cancer	823	IHC	99%	Associated with disease spread and poor outcome; an attractive targets for therapeutic manipulation

Sadun et al. [41]	Clin Cancer Res	2007	Breast and colorectal cancers	8 (breast), 11 (colorectal)	RT-PCR	100% (breast) Not consistent	A potential therapeutic target

Krambeck et al. [12]	Proc Natl Acad SCI USA	2006	Renal cell carcinoma	259	IHC	59.1%	B7-H4 is a useful prognostic marker for RRC patients

Sun et al. [8]	Lung Cancer	2006	Non-small-cell lung cancer	70	IHC	43%	Correlation with lower number of T cell infiltration

Simon et al. [37]	Cancer Res	2006	Colon, breast, lung, prostate, and ovarian cancers	1023 (confirmatory study : 200)	ELISA, IHC	—	Higher levels in endometrioid and serous histotypes than in mucinous histotypes in ovarian cancer

Tringler et al. [38]	Gynecol Oncol	2006	Ovarian cancer	125	IHC, WB	9% (mucinous), 100% (other histotypes and metastases)	A potential diagnostic marker or therapeutic target for ovarian cancer

Bignotti et al. [23]	Gynecol Oncol	2006	Ovarian serous papillary carcinoma	19	Microarray	—	Among the most highly overexpressed genes, indicating that B7-H4 is a candidate biomarker for early screening

Scalceda et al. [19]	Exp Cell Res	2005	Breast and ovarian cancers	19 (breast), 13 (ovarian)	RT-PCR, WB, IHC	100% (breast), 53.8% (ovarian)	A potential therapeutic target

Tringler et al. [33]	Clin Cancer Res	2005	Breast cancer	173 (primary), 246 (metastatic)	IHC	95.4% (primary), 97.6% (metastatic)	Correlation with negative progesterone receptor and HER-2/neu status, history of chemotherapy; no correlation with grade, stage

Choi et al. [4]	J Immunol	2003	Ovarian and lung cancers	22 (ovarian), 16 (lung )	IHC	85% (ovarian), 31% (lung)	A potential role in the evasion of tumor immunity

Notes: *Tumors from thyroid, esophagus, colon, pancreas, breast, liver, kidney, uterus, ovary, prostate and stomach. IHC: Immunohistochemistry; RT-PCR: Reverse transcription polymerase chain reaction; ELISA: Enzyme-linked immunosorbent assay; IP: Immunoprecipitation; WB: Western blot analysis; CA-125: Cancer antigen-125; CEA: Carcinoembryonic antigen.
